# Research on the construction of a sustainable scientific research capability evaluation model for university teachers based on the T-S fuzzy neural network

**DOI:** 10.1371/journal.pone.0313608

**Published:** 2025-02-10

**Authors:** Jia Wen, Pinhong Zeng

**Affiliations:** 1 Faculty of International Education, Yibin University, Yibin, China; 2 Faculty of Economics and Business Administration, Yibin University, Yibin, China; King Khalid University, SAUDI ARABIA

## Abstract

**Introduction:**

This study aims to enhance educational quality and academic standards by proposing a model based on critical research ability indicators to objectively evaluate the sustainable scientific research capabilities of university teachers.

**Methods:**

Using T-S fuzzy neural network technology, we developed an evaluation model to measure the sustainability of university teachers’ research capabilities. We collected data from 126 university teachers, using 90 samples for training and 36 for testing, to ascertain the model’s applicability and accuracy.

**Results:**

The T-S fuzzy neural network showcased exceptional learning efficiency and achieved a 98.15% accuracy rate in assessing the sustainable scientific research capabilities of university teachers, outperforming both Naive Bayes and BP neural networks in effectiveness.

**Conclusion:**

The research successfully constructs a T-S fuzzy neural network-based evaluation model for assessing the sustainable scientific research capabilities of university teachers. With high accuracy and broad applicability, this model is an effective tool for objectively evaluating university teachers’ research capabilities, clearly achieving the study’s objective.

## Introduction

In the realm of higher education, assessing the sustainable scientific research capabilities of university teachers is pivotal for enhancing educational quality and academic standards. A teacher’s research capability not only influences their personal career development but also significantly impacts student learning outcomes and the academic environment. Thus, effectively evaluating the sustainable research capabilities of university teachers is crucial for educational improvement.

Research refers to the exploration, study, and innovation of knowledge and information, including proposing new theories, designing and conducting experiments, collecting and analyzing data, and publishing new findings. The purpose of research is to create new knowledge, solve complex problems, develop new technologies or theories. In higher education institutions, teachers and researchers engage in research activities to advance the development and progress of their disciplines [1,2]. In recent years, researchers have conducted in-depth studies on the evaluation methods of university teachers’ research capabilities and established various evaluation models. For instance, evaluations of university teachers’ research capabilities often employ multidimensional systems based on publications, research projects, citation analysis, peer review, teaching contributions, social service, industry interaction, and the establishment of research teams [3]. Additionally, some studies focus more on aspects such as teachers’ professional knowledge, practice, participation, and self-management [4] and emphasize combining teachers’ teaching and research practices in evaluation methods, such as through Project-Oriented Learning (POL) to develop teachers’ capabilities in sustainability and research [5]. There are also studies proposing a university teaching staff capability evaluation model based on information entropy and uncertainty measurement, which includes aspects of professional quality, personality maturity, and driving ability [6].

However, traditional evaluation methods have their limitations. Firstly, traditional evaluation methods are limited in handling quantitative and qualitative data. Existing studies have highlighted that these methods face challenges in accommodating both quantitative and qualitative data evaluations [7]. Recent advancements in the performance assessment of complex systems incorporate multiple-criteria decision-making (MCDM) frameworks, showcasing their capability to manage diverse evaluation criteria effectively. For instance, scholars applied an MCDM model coupled with Self-Organizing Maps to assess various sustainable energy systems, illustrating the model’s depth and adaptability in complex scenarios [8]. This highlights the need for innovative approaches like the T-S fuzzy neural network proposed in our study, which aims to enhance evaluation accuracy by addressing the inherent subjectivity and integration challenges of traditional MCDM methods. This highlights the need for innovative approaches like the T-S fuzzy neural network proposed in our study, which aims to enhance evaluation accuracy by addressing the inherent subjectivity and integration challenges of traditional MCDM methods. Moreover, most current studies still focus on conceptual discussions of frameworks, lacking concrete implementation models [9,10]. This indicates that relying solely on traditional methods may not comprehensively reflect a teacher’s overall capabilities when evaluating their research ability. Secondly, traditional methods like bibliometrics, despite their advantages, also have multiple weaknesses [11]. As previously mentioned, some models overly depend on individual subjective judgments, making it difficult to integrate subjective and objective data. This indicates that a more comprehensive and integrated approach is necessary for evaluating the sustainable research capabilities of university teachers. Additionally, traditional evaluation methods may lead to increased subjectivity and arbitrariness in results, which could cause disputes across different disciplines [12]. In summary, traditional evaluation methods may not effectively assess the sustainable research capability of teachers from different disciplinary backgrounds and are challenging in combining subjective and objective data for complex evaluations.

Adopting the T-S fuzzy neural network as a new method for evaluating the sustainable research capability of university teachers can effectively overcome the limitations and bottlenecks encountered in existing literature.The T-S fuzzy neural network combines the advantages of fuzzy logic and neural networks. It handles complex and uncertain evaluation problems more effectively, providing more comprehensive, objective, and fair evaluation results [13,14]. In terms of specific evaluation methods, neural networks and fuzzy systems, with their powerful self-learning, self-organizing, and adaptive abilities, show their advantages in handling complex evaluation models. For example, the Takagi-Sugeno (T-S) fuzzy neural network, as a product of the combination of fuzzy theory and neural networks, effectively avoids issues such as a lack of learning ability, complex identification processes, and difficulty in optimizing model parameters while integrating the advantages of both methods.

Following the discussion on existing methods, the main innovation of this study lies in the development of a T-S fuzzy neural network-based evaluation model, specifically designed to assess the sustainable scientific research capabilities of university teachers. Unlike traditional assessment methods relied upon by previous studies, this model utilizes the advanced technology of the T-S fuzzy neural network, enabling more accurate and comprehensive handling of the uncertainties and complexities encountered during the evaluation process. By integrating the strengths of fuzzy logic and neural networks, our model not only enhances the objectivity and accuracy of the evaluations but also improves the handling of complex data, offering a new perspective and method for the objective evaluation of university teachers’ research capabilities. Furthermore, the effectiveness of this model is validated through empirical analysis, with its high accuracy in practical application further demonstrating significant improvements over existing methods.To further illustrate the unique advantages of the T-S fuzzy neural network compared to traditional methods, Table 1 contrasts several key performance metrics of our model against those of Naive Bayes and BP neural networks. This comparison underscores our model’s superior handling of complexities and its robustness in integrating subjective and objective data effectively.

**Table 1:** Comparative performance of evaluation models: T-S fuzzy neural network vs. traditional methods.

Feature	Naive Bayes	BP Neural Network	T-S Fuzzy Neural Network
Accuracy	Low	Medium	High
Handling Uncertainty	Poor	Moderate	Excellent
Adaptability to Complex Data	Low	Good	Very Good
Learning Capability	None	Yes	Yes
Integration of Fuzzy Logic	No	No	Yes
Subjectivity Handling	Poor	Good	Excellent

This study explores the factors affecting the sustainable scientific research capabilities of university teachers through literature reviews, questionnaire surveys, and interviews. Following a comprehensive literature review, we developed an interview outline and invited focus groups of doctoral students and graduate supervisors from various disciplines to discuss factors influencing sustainable scientific research capabilities. We selected research data from 126 faculty members of a university. Using the T-S fuzzy neural network, we constructed an evaluation model and compared its results with actual research outcomes to confirm the model’s accuracy and applicability.

The rest of this study is organized as follows: The first section provides a detailed overview of the theoretical basis for using BP neural networks and fuzzy neural networks to evaluate the sustainable scientific research capabilities of university teachers. This includes an explanation of the models’ structures, operational mechanisms, and their application in evaluations. The second and third sections elaborate on the process of developing a sustainable scientific research capability evaluation model based on fuzzy neural networks. This includes establishing an evaluation indicator system, conducting expert evaluations, and selecting and defining evaluation indicators. This part also presents the indicators for measuring university teachers’ sustainable scientific research capabilities and their weights, and examines the correlation between various factors and the evaluation results. The fourth section discusses the evaluation model used in this study, detailing the specific measurement and training processes. The fifth section evaluates the sustainable scientific research capabilities of university teachers based on the constructed model. This includes data acquisition, training results of the evaluation model, validation results, and an analysis of accuracy. Finally, the sixth section summarizes the main findings of the study, including the effectiveness and accuracy of the T-S fuzzy neural network in evaluating the sustainable scientific research capabilities of university teachers. It also discusses the study’s limitations, challenges encountered in practice, and directions for future research.

### Comparative study of BP and other neural networks in sustainable research capability evaluation for teachers

Neural networks are renowned for their mapping capability, generalization, robustness, and fault tolerance, which is why they have been widely applied in modeling reliable systems [15]. As a branch of artificial intelligence, artificial neural networks are used to simulate the human learning process, holding significant value in evaluating the sustainable scientific research capabilities of university teachers. The BP (Back Propagation) neural network, one of the most typical and widely utilized artificial neural networks, possesses strong computational power, information processing ability, learning capacity, and the ability to fit complex nonlinear relationships. These functional characteristics give it unparalleled advantages over traditional methods in analyzing the sustainable scientific research capabilities of university teachers.

The fundamental idea of using a BP neural network to establish a sustainable research capability evaluation model is as follows: elements of a teacher’s research capability are taken as input variables in data form, and indicators that can reflect their actual sustainable research capabilities are used as output variables to establish the neural network. By collecting a large number of samples to train the network and achieving the training objectives, relevant data are input for prediction. Decisions are then made based on the sustainable research capability status reflected in the prediction results.

However, this method has limitations when applied in reality. Directly using a BP neural network to establish such a sustainable research capability evaluation model not only requires acquiring a large amount of data affecting research capabilities and corresponding data on research capability levels for neural network learning but also demands precise scoring of each "capability factor"—that is, the evaluation indicators used as input variables. The evaluation and scoring of indicators related to research capabilities inherently involves strong subjectivity and ambiguity. Considering this, the fuzzy neural network, which combines fuzzy logic with artificial neural networks, may have greater applicability in evaluating sustainable research capabilities.

### Sustainable research capability evaluation model based on fuzzy neural networks

#### Basic principles and structure of fuzzy neural networks

Fuzzy neural networks combine the strong knowledge representation capability of fuzzy logic with the powerful learning capacity of neural networks. Fuzzy logic employs algorithms to represent human decision-making and evaluation processes [16], mitigating the uncertainty of subjective logic, and is especially suitable for evaluating complex systems like talent assessment.

The fundamental concept behind constructing fuzzy neural networks is to implement the fuzzy reasoning process through the structure of neural networks, while using the connection weights of the neural networks to express the parameters of fuzzy reasoning. Fuzzy neural networks can adjust weights via the BP algorithm to determine fuzzy rules and modify membership functions [17]. A typical fuzzy neural network structure includes four layers: the first layer is the input layer, the second and third layers are hidden layers, and the fourth layer is the output layer. In operation, signals enter the next layer through the input layer, then fuzzification and fuzzy reasoning are completed in the hidden layers, and finally, defuzzification is completed in the output layer, presenting the output results in numerical form [18].

### Advantages of the sustainable research capability evaluation model based on fuzzy neural networks

Firstly, fuzzy logic mitigates the ambiguity inherent in the evaluation process. Traditional evaluation methods rely on linear models to score various indicators, which are then aggregated according to their weights to produce an overall score [19–21]. This approach quantifies human subjective evaluations using simple mathematical models but remains imprecise. In the real world, many issues do not have absolute black or white answers. Attempting to solve these problems using traditional methods often results in answers that are neither precise nor efficient. Fuzzy logic addresses this by considering truth and falsehood (0 and 1) as extremes of possibility, with reality often lying in between. This allows fuzzy logic to handle decision-making problems containing vague information more effectively [22].

Fuzzy system theory is based on fuzzy sets. In classical set theory, a characteristic function of an item only takes two values: 0 and 1, representing "yes" or "no," such as evaluating a person as "excellent" or "not excellent." Fuzzy sets introduce the concept of a "fuzzy membership function," limiting the characteristic function values of items between 0 and 1, thus effectively resolving this issue. When applied to evaluating sustainable research capabilities, by transforming the scoring of various indicators and their weights into linguistic values through fuzzification, fuzzy logic can describe evaluators’ real assessments of university teachers’ sustainable research capability levels more accurately and scientifically than traditional evaluation methods.

Secondly, the network structure based on the BP algorithm can fit the nonlinear relationship between the scores of evaluation indicators and sustainable research capabilities, allowing for accurate predictions. BP neural networks, as the most typical type of artificial neural network, have strong computational and fitting capabilities. These networks are multi-layer feedforward networks characterized by forward signal transmission and backward error propagation. During forward transmission, input signals are processed layer by layer from the input layer through the hidden layers until they reach the output layer. If the desired output is not achieved, the process shifts to backward transmission, where the network adjusts weights and thresholds based on the prediction error. This continuous adjustment approximates the predicted output to the desired output. Unlike existing evaluation systems, methods based on artificial neural networks do not require any mathematical model or predefined weights for indicators. They rely on past experience and expert knowledge for learning, providing valuable references for evaluating the sustainable research capabilities of university teachers.

Neural networks, with their powerful self-learning, self-organizing, and adaptive abilities, are effective for establishing automated assessment models. Fuzzy systems, on the other hand, are highly applicable in dealing with uncertainty, imprecision of measurements, and other fuzzy issues. The T-S (Takagi-Sugeno) fuzzy system is an adaptive system that can automatically update and continuously correct the membership functions of fuzzy subsets. Fuzzy systems often face challenges such as lack of learning ability, complex identification processes, and difficulty in optimizing model parameters. In contrast, artificial neural networks possess self-learning, self-organizing, and adaptive capabilities with strong nonlinear processing abilities. The T-S (Takagi-Sugeno) fuzzy neural network combines the advantages of both methods, effectively avoiding problems like lack of learning ability, complex identification processes, and difficulty in optimizing model parameters [23].

## Evaluation of sustainable research capabilities based on fuzzy neural networks

**Fig 1:**
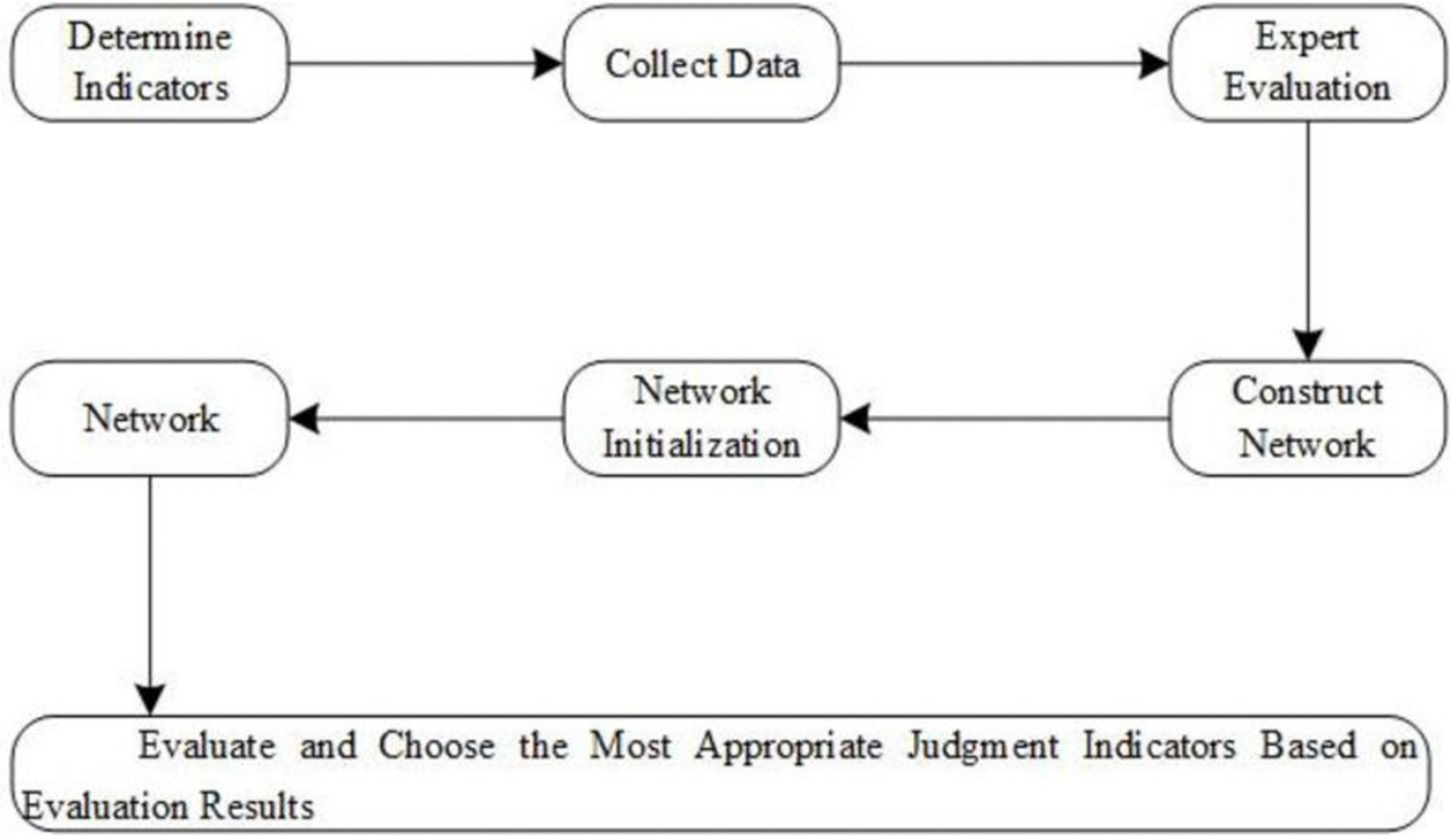
Evaluation of sustainable research capabilities based on fuzzy neural networks.

The fundamental approach to establishing an evaluation of sustainable research capabilities based on fuzzy neural networks involves several steps. First, collect sufficiently typical sample data and establish a comprehensive and reasonable indicator system. Next, determine the network structure and use the scores of various indicators after fuzzification to obtain corresponding membership degrees as inputs. The scores based on actual sustainable research capabilities are used as outputs. Finally, the trained network processes the relevant data of candidate teachers to obtain numerical scoring results. Based on these scores, the sustainable research capabilities of university teachers are assessed. The sustainable research capability evaluation system based on fuzzy neural networks is illustrated in Fig 1.

### Establishment of the evaluation indicator system

Establishing a comprehensive, reasonable, and operable evaluation indicator system is essential, whether using traditional evaluation methods or fuzzy neural networks. Methods for designing such a system include key figure analysis, hierarchical methods, and expert surveys.

### Expert evaluation

Eight senior professors from different disciplines evaluated 126 teachers based on the established indicator system. To ensure comprehensive learning by the neural network, the training data was sufficiently large. They also made overall assessments of the teachers’ actual sustainable research capabilities. The collected evaluation results were used as training data.

## Evaluation indicators and analysis of related factors

### Selection of evaluation indicators

When constructing an evaluation indicator system for the sustainable research capabilities of university teachers, it is essential to consider multiple dimensions to ensure comprehensiveness and accuracy. The following evaluation indicators were selected for this study:

First, Research Output. Research output is the core indicator for evaluating a teacher’s research capability. This includes the number and quality of published papers (such as peer reviews, impact factors, etc.), the number and quality of research projects, citation analysis, etc. These indicators directly reflect the outcomes of a teacher’s research activities and their academic influence [3].

Second, Teaching Contribution. Teaching is one of the fundamental responsibilities of university teachers. It involves the process of imparting knowledge and skills, including preparing course plans, teaching course content, assessing students’ learning outcomes, etc. The goal of teaching is to impart knowledge, develop skills, inspire students’ interest, and foster critical thinking. Teachers not only convey existing knowledge in the teaching process but may also encourage students to explore and research [24,25]. In many universities and colleges, teachers often undertake both research and teaching responsibilities, and the relationship between the two can be complementary. Research can enhance teaching: new knowledge and experiences gained from research can enrich their teaching content, allowing students to access cutting-edge research findings and practices [26,27]. Similarly, teaching can also promote research: questions raised and feedback and discussions in teaching activities can sometimes inspire new research ideas and directions [28,29].

Third, Practical Interaction. Interaction with the real world, such as collaborative research, consulting, knowledge support, etc., can reflect the practical application value of a teacher’s research outcomes. This interaction helps to transform theoretical research into practical applications and is an important manifestation of research capability [3].

Fourth, Research Team. The ability to establish and maintain an effective research team is also an important aspect of evaluating a teacher’s research capability. Team-building ability reflects a teacher’s leadership and collaborative spirit [3].

Fifth, Research Innovation. A teacher’s innovation in research, including adopting new research methods, developing new theories or technologies, etc. Innovation is the core of research, reflecting the teacher’s originality and foresight [30].

Sixth, Organizational Rewards. The reward mechanism of schools for teachers’ research output is also an important consideration. This includes research funding, bonuses, promotion of professional titles, etc. These rewards can motivate teachers to invest more effort in research work [31].

Seventh, Attention to Cutting-Edge Literature. Whether teachers regularly pay attention to and cite cutting-edge literature reflects their ability to keep up with academic updates. Keeping abreast of the latest research trends is an important part of research work, helping to enhance the relevance and innovation of research [32].

Eighth, Self-Learning Ability. A teacher’s self-learning ability, especially in emerging fields, is an important manifestation of their research capability. This not only helps teachers keep their knowledge updated but also promotes the development of interdisciplinary research [33].

Ninth, Academic Honors. A teacher’s academic honors, such as awards and honorary titles received, are also an important aspect of measuring their research capability. This reflects the teacher’s status and influence among peers [34].

In summary, constructing an evaluation system for the sustainable research capabilities of university teachers requires considering multiple aspects. These indicators not only cover the teacher’s research output and quality but also include teaching, research innovation, industry interaction, and more, thereby offering a comprehensive assessment of teachers’ research capabilities, as detailed in Table 2. When constructing the evaluation system, these diverse aspects should be considered to ensure the comprehensiveness and fairness of the evaluation.

**Table 2:** Evaluation indicator system for sustainable research capability of teachers.

Indicator	Description
Research Output	Based on the research documents of each teacher’s institution, the number of articles published in journals of various levels at their respective schools and the number of funded projects at different levels (national, provincial/ministerial, and municipal/bureau level, with weights determined by expert method as 0.45, 0.35, 0.20 respectively) are counted. The weights of these three indicators are determined through the expert method (A+ journal weight is 0.45, A journal is 0.3, B journal is 0.15, C journal is 0.1; similar adjustments are made for different universities). The final weight for the number of articles is 0.55, and for research projects is 0.45.
Teaching Contribution	Measured by the number of honors and awards received in teaching (with weights for national, provincial/ministerial, and municipal/bureau level honors determined by expert method as 0.45, 0.35, 0.20 respectively).
Practical Interaction	Measured by the frequency of interaction between teachers and real-world organizations, such as the frequency of collaborative research, consulting, knowledge support.
Research Team	Measured by the teacher’s involvement in research teams (0 represents no participation or leadership in any research team), with higher values indicating participation or leadership in more research teams.
Research Innovation	Judged by experts based on the novelty of methods used in the teacher’s published papers. If a method in a paper is considered cutting-edge in the field, it is assigned a value of 1, otherwise 0, and these are cumulatively calculated.
Organizational Rewards	Judged based on the relevant documents of the teacher’s institution, i.e., the level of funding and rewards stipulated for teachers in the documents. Weights are determined through expert judgment, and the product of the two is used to calculate the Organizational Reward Index, which is then normalized for analysis.
Cutting-Edge Literature	Measured by the frequency with which teachers read cutting-edge literature.
Self-Learning Ability	Measured by the difficulty level for teachers in mastering new methods and knowledge.
Academic Honors	Measured by the number of honors and awards received in research (with weights for national, provincial/ministerial, and municipal/bureau level honors determined by expert method as 0.45, 0.35, 0.20 respectively).

### Identifying significant factors affecting the sustainable research capabilities of university teachers

The factors influencing the sustainable research capabilities of university teachers, gathered through qualitative research methods such as interviews and self-assessments, are subjectively biased and can only serve as a preliminary delineation of scope. After establishing measurement standards, this study analyzes these factors and their correlation with the sustainable research capabilities of university teachers in a quantitative manner, referring to existing research approaches [35]. This allows for the identification of significantly correlated factors while filtering out weakly related ones, ensuring research accuracy. Using the scores obtained from the T-S fuzzy neural network to evaluate the sustainable research capabilities of university teachers, this study employs SPSS 27.0 for Spearman correlation analysis.

#### Principles and results of correlation testing

The Spearman correlation coefficient between two random variables X and Y is denoted as ρ (rho). Its calculation formula is:

$$r=1-\frac{6\sum_{i-1}^{n}\;D_{i}^{2}}{n(n^{2}-1)}$$ (1)

where:

$$\sum_{i=1}^{n}\;D_{i}^{2}=\sum_{i=1}^{n}\;{\left(U_{i}-V_{i}\right)}^{2}$$ (2)

Here, X represents the sustainable research capability evaluation scores, Y represents the 9 significant influencing factors, U_i_ and V_i_ are the ranks assigned to X and Y after sorting, and n is the sample size.

The results show strong correlations between the sustainable research capabilities of university teachers and the following factors: research output (P<0.001), teaching contribution (P<0.05), practical interaction (P<0.05), research team (P<0.01), research innovation (P<0.01), organizational rewards (P<0.001), attention to cutting-edge literature (P<0.001), self-learning ability (P<0.001), and academic honors (P<0.01), all showing significant correlations (P<0.05).

## Evaluation model

### T-S fuzzy neural network

This study employs the T-S fuzzy neural network, which has strong adaptive capabilities, able to automatically update and continuously correct fuzzy membership functions. The T-S fuzzy neural network comprises an antecedent network and a consequent network, forming a nonlinear fuzzy inference model characterized by its computational simplicity, ease of integration with adaptive methods, and suitability for mathematical analysis [36]. Its input consists of the evaluation scores of various indicators of university teachers’ research capabilities, while the output is the assessed level of sustainable research capabilities. Thus, the network used in this study considers multiple inputs and a single output.

The fuzzy rule form of the T-S fuzzy system is shown in Eq (3):

$$R^{i}:\;if\;x_{1}\;\mathrm{is}\;A_{1}^{i},x_{2}\;\mathrm{is}\;A_{2}^{i},\cdots ,x_{k}\;\mathrm{is}\;A_{k}^{i},\;\mathrm{then}\;y_{i}=p_{0}^{i}+p_{1}^{i}x_{1}+\cdots +p_{k}^{i}x_{k}$$ (3)

Here, $A_{j}^{i}$ is a fuzzy set defined in the domain of x_j_, representing the ith linguistic value of x_j_ and the set of the jth indicator corresponding to the ith evaluation; $p_{j}^{i}(i=1,2,\cdots ,n;j=1,2,\cdots ,k)$ represents the parameters of the fuzzy system; y_i_ is the output obtained according to the fuzzy rules, which is the overall score of each indicator of sustainable research capability of university teachers. The input part is the ’if’ part, and the output part is the ’then’ part.

#### Antecedent network

The network structure of this system is divided into four layers: input layer, fuzzification layer, fuzzy inference layer, and output layer. The input layer has the same number of nodes as the dimension of the input vector, i.e., the number of evaluation indicators. The fuzzification layer uses membership functions to fuzzify the input values and obtain the membership values μ, representing the degree to which each indicator belongs to each evaluation.

1. Input Layer. This is the input x_i_ in the network, as shown in Eq (4).
$$x_{i}=(x_{1},x_{2},x_{3},\cdots ,x_{n})i=1,2,\cdots ,n$$ (4)
  Here, n is the number of input nodes.
2. Fuzzification Layer. The membership degree $u_{j}^{i}$ of each input variable is calculated using the membership function, as shown in Eq (5).
$$u_{j}^{i}=exp\;\left[-{\left(x_{i}-c_{j}^{i}\right)}^{2}/b_{j}^{i}\right]\;j=1,2,\cdots ,m$$ (5)
  Here, $c_{j}^{i}$ is the center of the membership function, $b_{j}^{i}$ is the width of the membership function, and m is the number of input parameters.
3. Fuzzy Rule Calculation Layer. The adaptability ω^j^ of each rule is calculated using the fuzzy product formula, as shown in Eq (6).
$$\omega^{j}=u_{j}^{1}(x_{1})\times u_{j}^{2}(x_{2})\times \cdots \times u_{j}^{n}(x_{n})$$ (6)
4. Output Layer. The output results of the antecedent network are defuzzied to calculate the weights $\bar{\omega^{j}}$ of each attribute, as shown in Eq (7).
$$\bar{\omega^{j}}=\frac{\omega^{j}}{\sum_{j=1}^{n}\;\omega^{j}}$$ (7)

### Consequent network

1. Input Layer. This refers to the input x_i_ in the network, whose function is to transmit the input variables to the fully connected layer.
2. Fully Connected Layer. It receives the input variables from the input layer and implements learning of the input variables using Eq (8) to obtain the output result $x_{i}^{\left(2\right)}$.
$$x_{i}^{\left(2\right)}=p_{j}^{0}+\sum_{i=1}^{n}\;p_{j}^{i}x_{i}$$ (8)
  Here, $p_{j}^{i}$ represents the neural network coefficients.
3. Output Layer. It receives the weights $\bar{\omega^{j}}$ from the antecedent network and the output result $x_{i}^{\left(2\right)}$ from the consequent network. The final output result Eout^j^ of the T-S fuzzy neural network is calculated using Eq (9).
$${\mathrm{Eout}}^{j}=\sum_{j=1}^{n}\;\bar{\omega^{j}}x_{i}^{\left(2\right)}$$ (9)

### Evaluation model initialization

To avoid prolonged training time due to the presence of singular sample data, it is necessary to normalize the training data first. The normalization formula is shown in Eq 10:

$$x_{i}=\frac{X_{i}-minX_{i}}{maxX_{i}-minX_{i}}$$ (10)

Based on the T-S fuzzy neural network theory, a model for evaluating the sustainable research capability of university teachers is established using MATLAB R2022b. According to the research capability evaluation indicators, the model’s input data is 9-dimensional, and the output data is 1-dimensional. Ten sets of coefficients p_0_∼p_9_ are selected according to Eq (5). To determine the number of intermediate layers and learning efficiency of the neural network, pre-training is conducted with intermediate layers set at 40, 45, and 50, and learning rates at 0.03, 0.05, and 0.07. Comparing the pre-training results, the smallest error in the T-S fuzzy neural network training results is achieved when the number of intermediate layers is 45 and the learning rate is 0.05. Therefore, the neural network structure constructed in this paper is determined to be 9-45-1.

After determining the network structure, it is necessary to initialize the fuzzy system parameters and membership function parameters of the fuzzy neural network. Additionally, the maximum number of evolutions and the learning rate of the network need to be preset.

### Evaluation model training

Combining objective data and expert assessments, the maximum deviation values of the 9 evaluation indicators are used as inputs. The model outputs the evaluation results of university teachers’ sustainable research capabilities, which are then compared with the experts’ scoring results to verify the effectiveness of the model.

Suitable groups of normalized maximum deviation values and training results are selected to train the T-S fuzzy neural network. The maximum deviation values of the indicators serve as the inputs to the model, while the scoring results of experts on the level of sustainable research capabilities, i.e., the expert evaluation results, serve as the model’s outputs. The learning rate of the neural network is set to 0.05, and it is trained for 5000 iterations using the mean square error as the error calculation function of the model, shown in Eq (11).


$$e=\frac{1}{2}{\left(y_{d}-y_{e}\right)}^{2}$$ (11)

Here, y_d_ is the desired output of the network; y_e_ is the actual output of the network. During the training process, the coefficients are adjusted, as shown in Eqs (12) and (13).


$$p_{j}^{i}(m)=p_{j}^{i}(m-1)-\alpha \frac{\partial e}{\partial p_{j}^{i}}$$ (12)

$$\frac{\partial e}{\partial p_{j}^{i}}=\frac{(y_{d}-y_{e})\omega^{j}}{\sum_{i=1}^{n}\;\omega^{j}x_{i}}$$ (13)

Here, $p_{j}^{i}$ represents the neural network coefficients, α is the learning rate of the network, ω^j^ is the product of membership degrees, and x_i_ is the network input parameter. The parameter adjustment is shown in Eqs (14) and (15).


$$c_{j}^{i}(m)=c_{j}^{i}(m-1)-\beta \frac{\partial e}{\partial c_{j}^{i}}$$ (14)

$$b_{j}^{i}(m)=b_{j}^{i}(m-1)-\beta \frac{\partial e}{\partial b_{j}^{i}}$$ (15)

**Fig 2:**
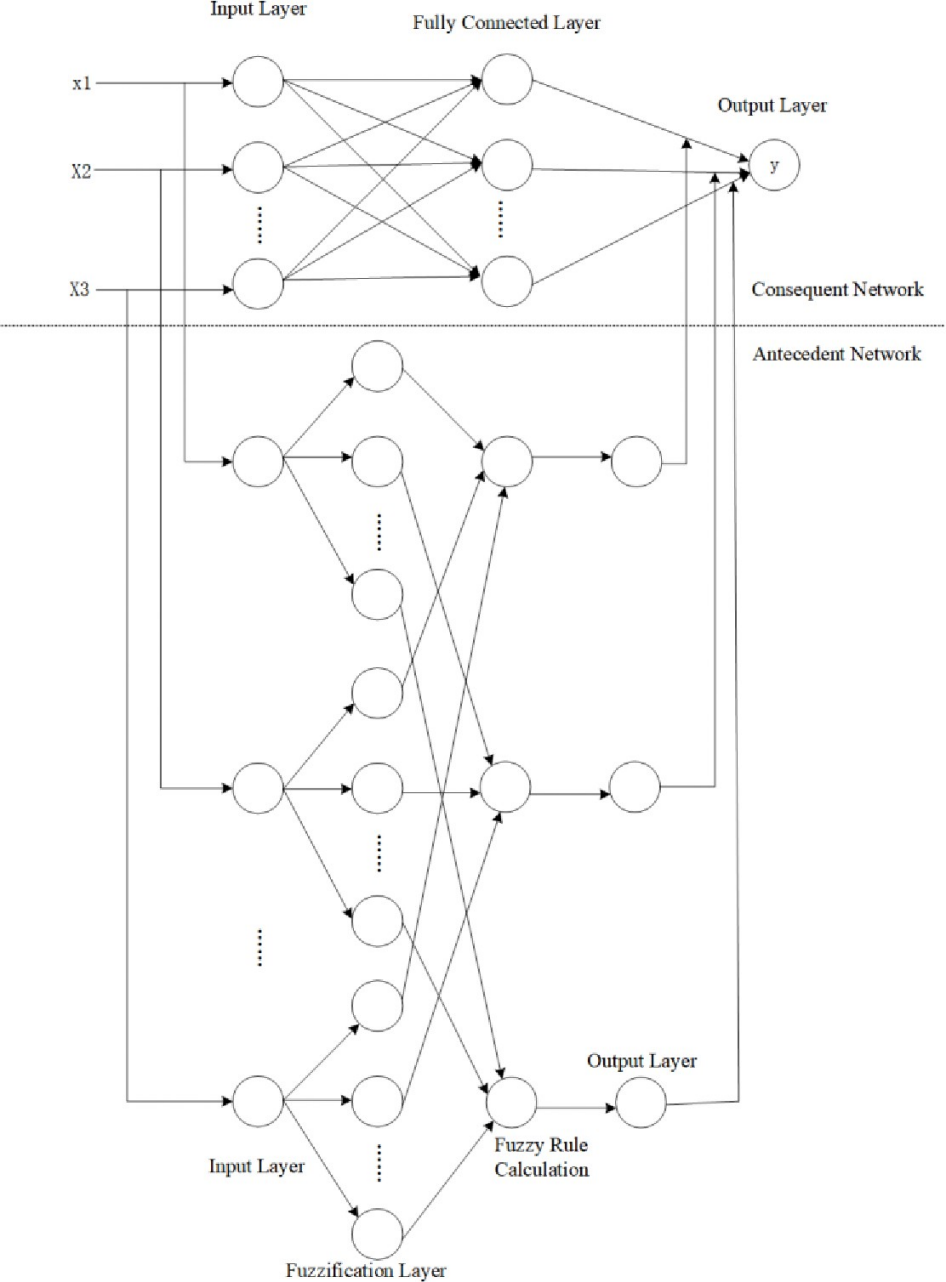
T-S fuzzy neural network topology.

Here, $c_{j}^{i}$ is the center of the membership function, $b_{j}^{i}$ is the width of the membership function, and β is the coefficient of the neural network. After training the network through multiple adjustments of coefficients and parameters using the training data, the T-S fuzzy neural network-based evaluation model for sustainable research capability of university teachers is obtained. The topology of the model is shown in Fig 2.

## Evaluation of university teachers’ sustainable research capabilities

### Data acquisition

To obtain the indicator data and expert evaluation data necessary for training and validating the sustainable research capability evaluation model, interviews and surveys were conducted with 150 university teachers from both key and non-key universities in China. Relevant indicator data over the past 5 years were collected, covering an age range of 30 to 50 years and ranks from lecturers to professors. Additionally, 8 full professors provided comprehensive scoring.

The purpose of the interviews was primarily to gather data on practical interaction frequency, reading of cutting-edge literature, and self-learning capabilities of university teachers. For indicators such as research output, teaching contribution, research team, research innovation, organizational rewards, and academic honors, teachers provided objective data based on actual situations.

It is important to note that consent was obtained from the participants at the outset of data collection, and the study committed to keeping personal data and privacy confidential. The research design thoroughly considered principles of safety and fairness, ensuring that the content would not harm or pose risks to participants. Recruitment of subjects was based on voluntariness and informed consent, with maximal efforts made to protect participants’ rights and privacy, avoiding any conflict of interest in the research content and results.

After collecting interview and survey data, 8 professors from various disciplines evaluated the scores of teachers in closely related disciplines across various indicators. The collected indicator data and expert evaluation results were then verified for completeness and accuracy and preserved. Ultimately, a dataset of various indicators and expert evaluation results for 126 teachers was compiled. Based on the 9 indicators of university teachers’ sustainable research capabilities and considering the data situation of university teachers over the past 5 years, sustainable research capabilities were classified into 5 levels: Level 1, Level 2, Level 3, Level 4, and Level 5, each corresponding to five scoring intervals: [100∼89.5), [89.5∼79.5), [79.5∼69.5), [69.5∼59.5), [59.5∼0].

### Training results of the evaluation model

**Fig 3:**
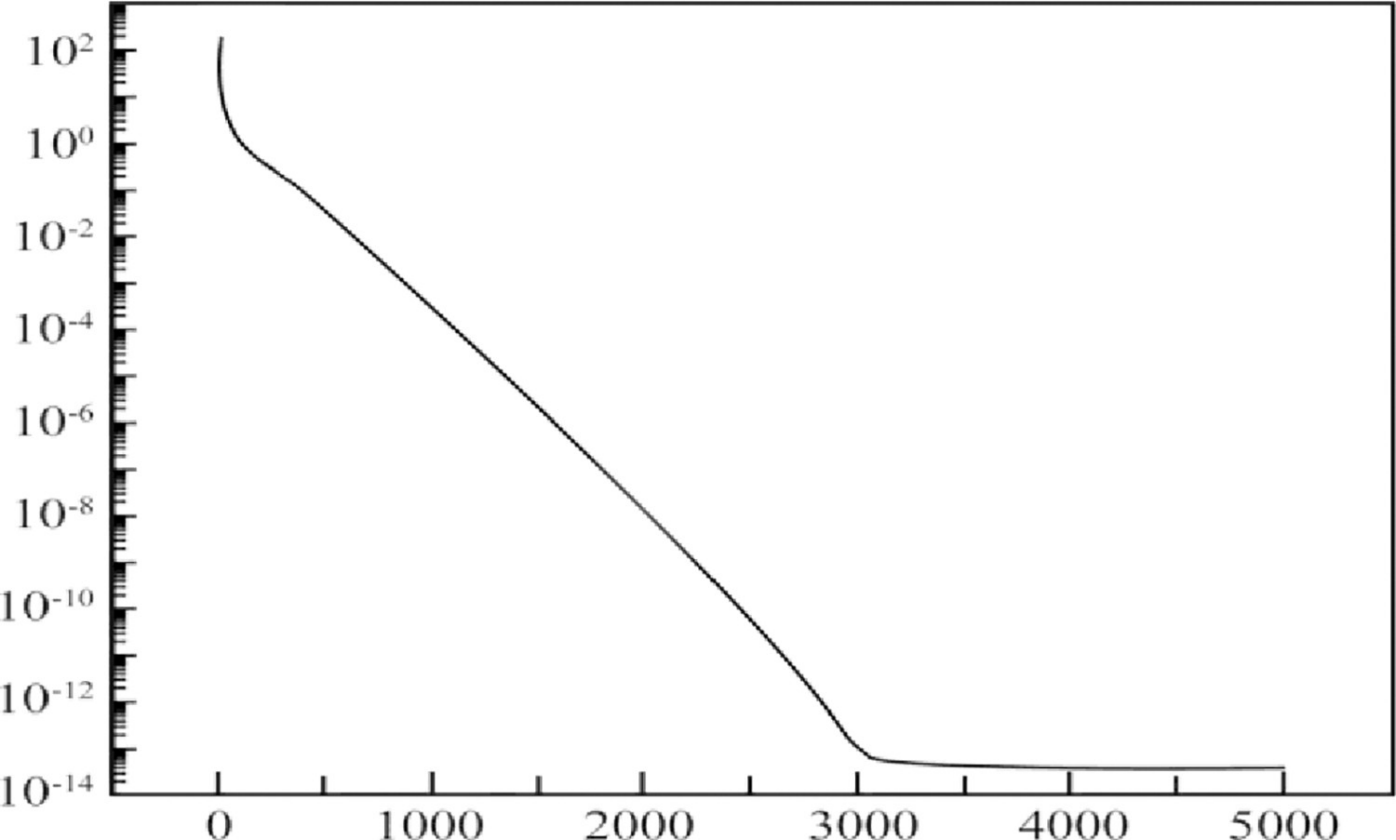
MSE of network training.

Randomly selecting 90 sets of normalized indicator values and evaluation results, the T-S fuzzy neural network was trained. The normalized values of the 90 sets of indicators were used as model inputs, while the scores and evaluation results given by experts on the sustainable research level of teachers served as model outputs. The mean square error during the network training process is illustrated in Fig 3.

As shown in Fig 3, during the initial 3000 iterations, the mean square error of the network decreases rapidly and then stabilizes. After 5000 iterations, the network’s mean square error reaches a magnitude of 10^−3^. From the training mean square error, it can be inferred that the T-S fuzzy neural network exhibits good convergence in the learning process of evaluating university teachers’ sustainable research capabilities. The evaluation model established using this network can quickly meet convergence requirements.

**Fig 4:**
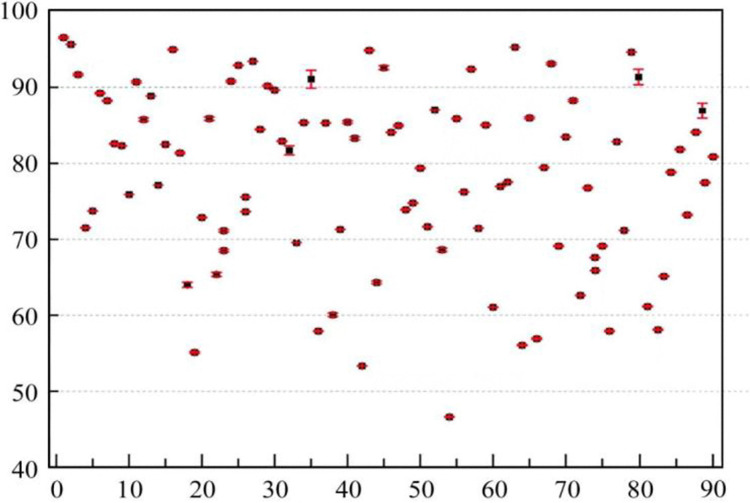
Evaluation results of the training set.

Fig 4 presents the model evaluation results for the training set of the sustainable research capability evaluation model for university teachers. Overall, the model evaluation results show good consistency with the training outcomes, except for four training set samples (numbers 33,55,80 and 87), which display significant discrepancies between model evaluation results and assessment outcomes. The training outcomes indicate that the sustainable research capability evaluation model for university teachers based on the T-S fuzzy neural network achieves favorable training effects.

### Validation results of the evaluation model

**Fig 5:**
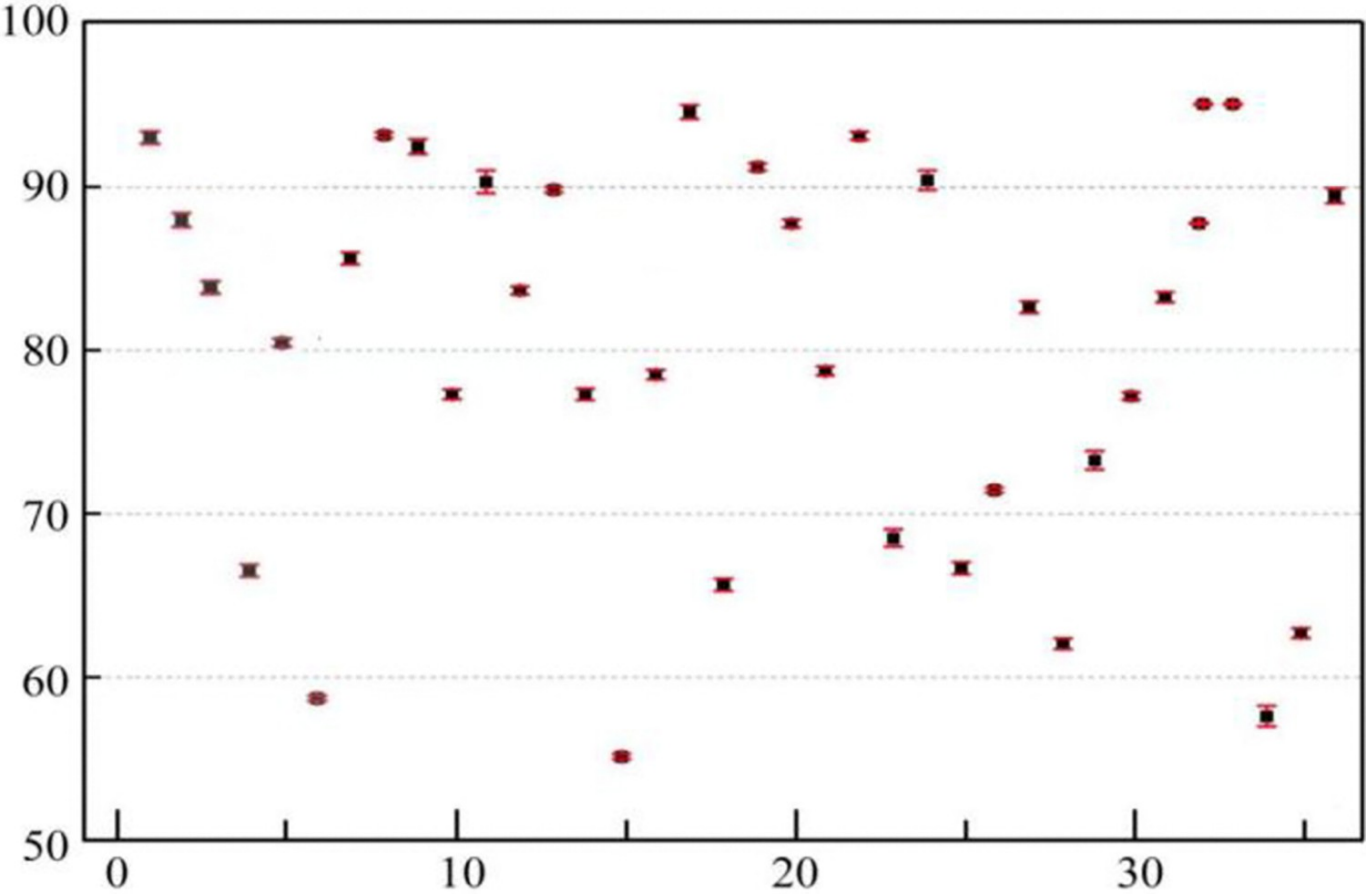
Evaluation results of the test set.

The remaining 36 datasets were input into the trained evaluation model as a test set, and the output results were then denormalized as shown in Fig 5. Analyzing the length of the error bars in Fig 5, it can be observed that the relative error in the model evaluation results for the test set has increased, but the magnitude of the increase is low. Overall, the evaluation model for sustainable research capabilities of university teachers demonstrates minor errors compared to expert evaluation results for the test set, achieving the goal of evaluating the sustainable research capabilities of university teachers. This indicates that the T-S fuzzy neural network possesses good learning and generalization capabilities.

**Fig 6:**
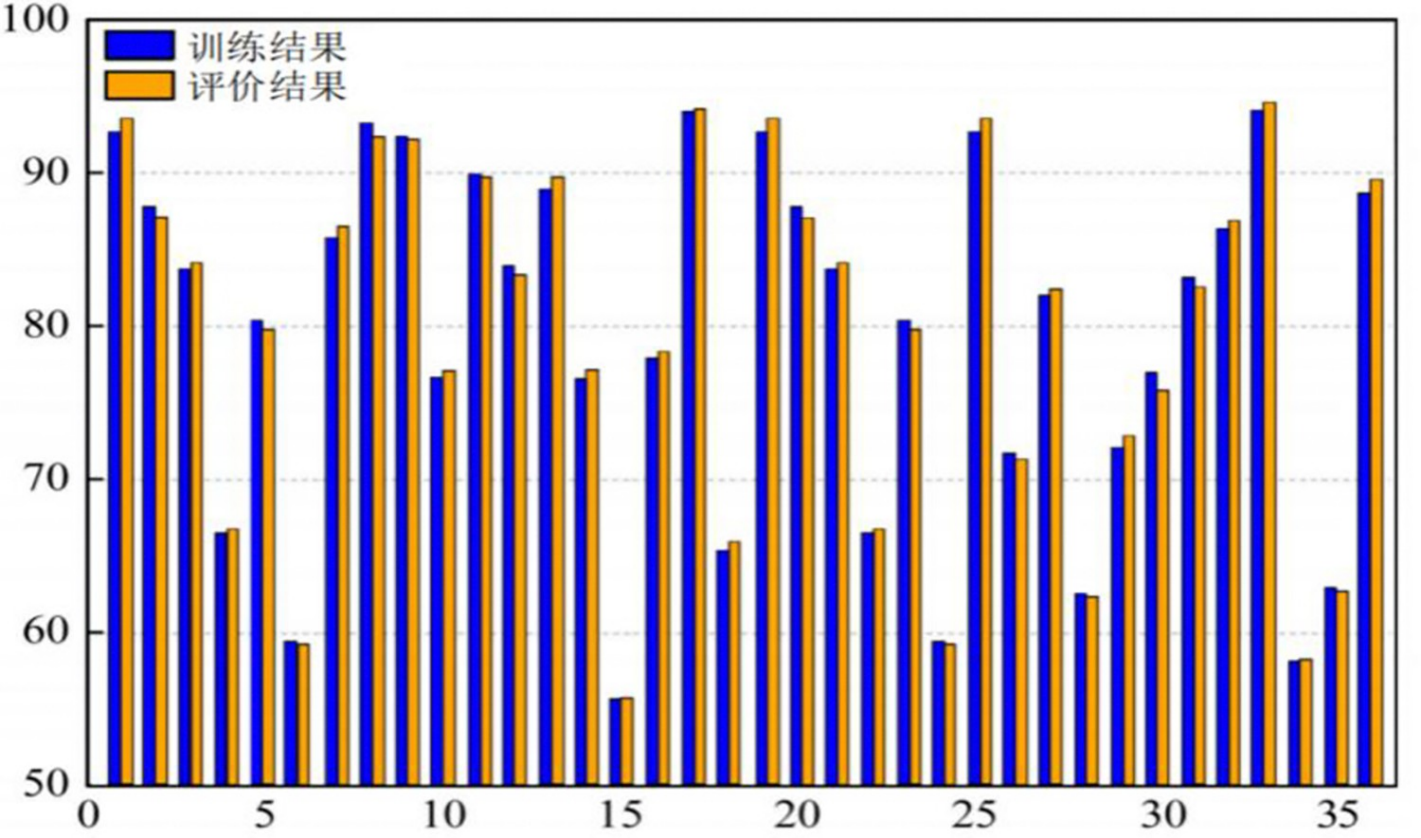
Evaluation results of sustainable research capabilities of 36 university teachers.

Statistical analysis was conducted on the training results of the sustainable research capabilities of 36 university teachers and the evaluation results obtained from the sustainable research capability evaluation model, as illustrated in Fig 6.

From Fig 6, it is evident that the evaluation results of the sustainable research capabilities of the 36 university teachers are distributed across all 5 levels. The absolute error between the model evaluation results and the expert evaluation results is less than 1, satisfying the tolerance requirements set for the scoring intervals. Among the 36 university teachers, 15 were evaluated as Level 1 for sustainable research capability, 9 as Level 2, 5 as Level 3, 3 as Level 4, and 4 as Level 5.

The evaluation results show a negative correlation with the tenure of the 36 university teachers. Among these teachers, those who have recently joined the faculty are all above Level 2, whereas the 7 teachers evaluated at Levels 4 and 5 are more experienced. The 15 teachers evaluated at Level 1 are all young teachers who have recently joined the faculty. This could be due to the pressure of a "up or out" policy, necessitating continuous effort to meet the school’s research assessment conditions to remain. Among them, 6 teachers have published multiple papers in top-tier core journals within their fields; the 9 teachers evaluated at Level 3 are all young associate professors, who need to further enhance their research capabilities for promotion to full professorship; the 5 teachers evaluated at Level 4 are relatively older and all hold the position of associate professor; the other 7 more senior teachers may have been evaluated lower in sustainable research capability due to their age and their professional ranks being essentially fixed, yet 5 of these teachers have demonstrated a high level of research performance.

### Accuracy analysis

The accuracy of the T-S fuzzy neural network’s evaluation, denoted as EA, was calculated by substituting the training results and model evaluation results into Eq (16):

$$EA=\frac{\sum_{j=1}^{N}\;1-|{\mathrm{Tout}}^{j}-{\mathrm{Eout}}^{j}|/{\mathrm{Tout}}^{j}}{N}$$ (16)

In this formula, N represents the number of data groups for evaluation (N = 30); Tout ^j^ and Eout ^j^ are the results of the j^th^ group’s training evaluation and the neural network evaluation output after denormalization, respectively.

To verify the superiority and accuracy of the T-S fuzzy neural network in evaluating the sustainable research capabilities of university teachers, and to compare it with modeling methods applied in performance evaluation, evaluation models were established using Naive Bayes and BP neural networks. The accuracies of the three evaluation models, calculated using Eq (14), in ascending order of accuracy were Naive Bayes, BP neural network, and the T-S fuzzy neural network, with accuracies of 69.53%, 93.68%, and 98.15%, respectively. The T-S fuzzy neural network achieved the highest accuracy among the three methods, indicating its strong adaptability in evaluating the sustainable research capabilities of university teachers.

The evaluation model established in this article for assessing the sustainable research capabilities of university teachers compensates for potential errors introduced by subjective scoring from different experts, while considering various indicators that influence university teachers’ sustainable research capabilities. The T-S fuzzy neural network-based evaluation model has effectively served as a valuable tool in evaluating the sustainable research capabilities of university teachers.

### Robustness test

Following the approaches of references [37–39], this study conducted robustness tests on the T-S fuzzy neural network model. Table 3 shows the maximum allowable delay of the system under different μ values. Comparing the results from different references, our model demonstrates superior robustness. Specifically, when μ = 0, the maximum allowable delays reported in references [37–39] are 0.143713,0.143364, and 0.142823, respectively. As the μ value increases, the maximum allowable delay decreases. For instance, when μ = 1.2, the maximum allowable delays drop to 0.023535,0.010855, and 0.035028, respectively. In contrast, the results from reference [39] consistently show higher maximum allowable delays across all μ values, indicating a more conservative approach to delay tolerance while still maintaining system stability at higher μ values. Additionally, when the μ value is unknown, the maximum allowable delay further decreases, suggesting that the system must adopt more conservative strategies to maintain stability in the absence of μ value information.

**Table 3:** Maximum allowable delay of the system under different μ values.

Reference	0	0.1	0.5	0.9	1.2	Unknown μ
[37]	0.143713	0.082407	0.090583	0.068272	0.023535	0.049299
[38]	0.143364	0.090684	0.063459	0.031833	0.010855	0.040234
[39]	0.142823	0.113505	0.092192	0.067235	0.035028	0.016765

**Fig 7:**
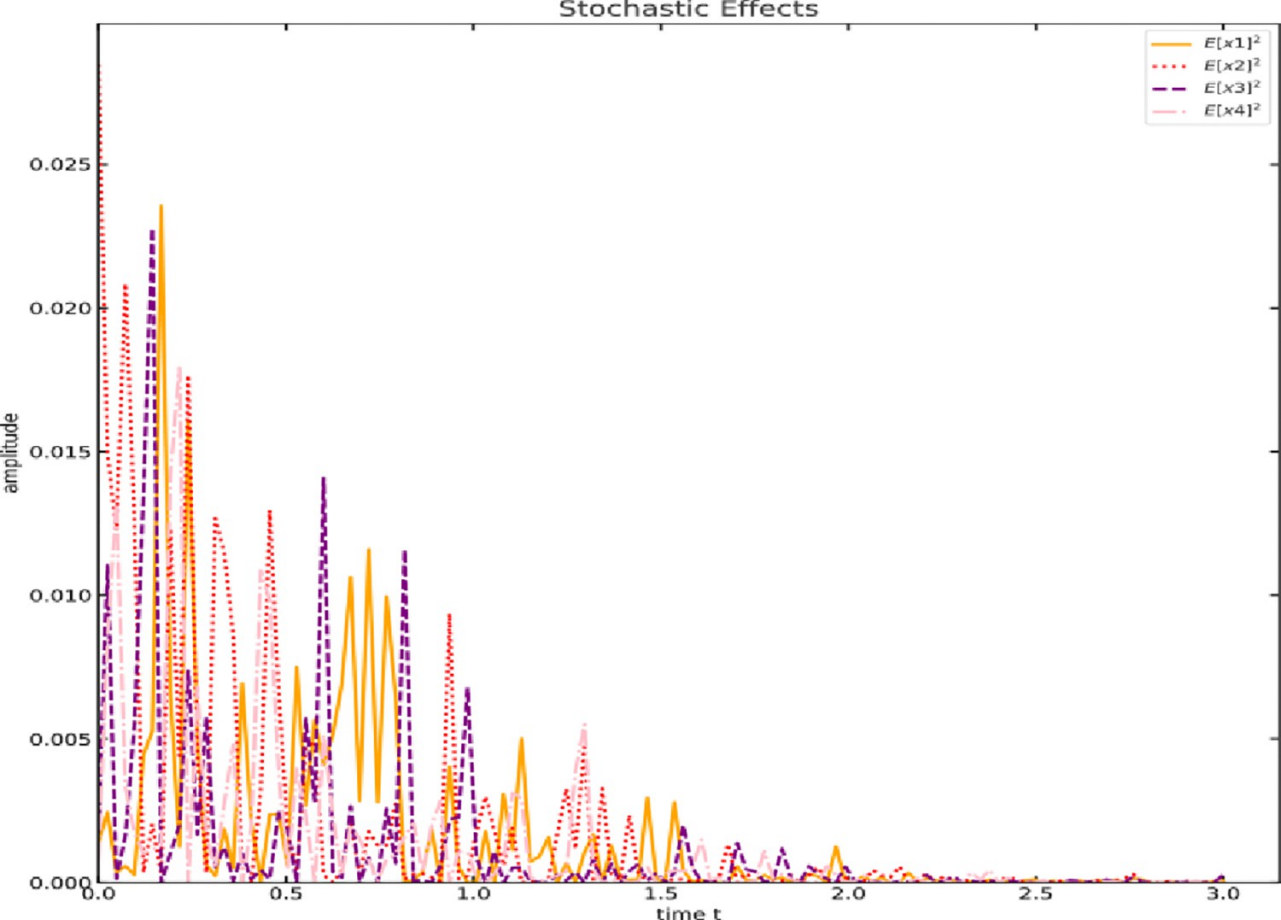
Convergence of system state variables over time.

Fig 7 illustrates the convergence of system state variables over time. The trajectories of four different state variables provide a clear view of the system’s stability and dynamic behavior. At t≈0, all state variables exhibit significant fluctuations, reflecting the system’s adaptation process to initial conditions and external disturbances. As time progresses, the amplitude of all state variables gradually decreases and eventually approaches zero, indicating the system’s ability to effectively suppress disturbances and return to equilibrium under the current parameter settings. From approximately t = 1.0, the fluctuations of the state variables significantly diminish, indicating the system has reached a stable state.

Specifically, E[x_1_(t)]^2^ and E[x_2_(t)]^2^ show larger fluctuations during the convergence process, likely due to greater external disturbances or stronger initial condition impacts. In contrast, E[x_3_(t)]^2^ and E[x_4_(t)]^2^ exhibit smaller fluctuations, indicating higher robustness within the system and a quicker adaptation to changes, thus returning to equilibrium more swiftly. These state variables ultimately converge to zero, confirming the system’s robustness under current conditions. If certain variables fail to converge or continuously oscillate, further adjustments to system parameters may be necessary to enhance stability. Fig 7 effectively demonstrates the system’s dynamic behavior and stability, further validating the robustness of the proposed model under various conditions.

## Conclusion and discussion

### Conclusion

This study developed a sustainable research capability evaluation model for university teachers based on the T-S fuzzy neural network, thoroughly exploring the multidimensional evaluation of university teachers’ research capabilities. By considering nine key indicators, including research output, teaching contribution, practical interaction, research team, research innovation, organizational rewards, attention to cutting-edge literature, self-learning ability, and academic honors, this study conducted an in-depth analysis using the T-S fuzzy neural network model, leading to several key findings:

1. The T-S fuzzy neural network model demonstrated high accuracy (98.15%) in evaluating the sustainable research capabilities of university teachers. Compared to traditional methods such as the Naive Bayes approach and the BP neural network, the T-S fuzzy neural network showed greater adaptability and effectiveness in this study. This high level of accuracy underscores the model’s potential to enhance empirical evaluation methodologies, especially in dynamically changing academic environments. By combining the strengths of neural networks and fuzzy systems, the T-S fuzzy neural network is capable of complex processing of both subjective and objective data, thus offering a more nuanced and robust tool for academic evaluations than previously available. The training and testing results of the model indicate its suitability for evaluating the sustainable research capabilities of university teachers.
2. Through empirical analysis of 126 teacher samples, this study not only verified the model’s effectiveness but also revealed the diversity and complexity of university teachers’ sustainable research capabilities. This diversity is critical, as it highlights the varied research environments and different academic demands across disciplines.By integrating expert interviews and literature reviews, nine indicators were selected as training metrics, forming an evaluation indicator system for sustainable research capabilities of university teachers. The integration of these diverse metrics provides a comprehensive view that respects individual differences and promotes a balanced approach to faculty evaluation.
3. The results of this study provide university administrators with a scientific and objective tool for evaluating teachers’ research capabilities, aiding in the more accurate identification and development of teachers with high research potential. This tool allows for strategic planning in faculty development and resource allocation, aligning institutional goals with individual capabilities. It also offers guidance for individual teachers’ career development and academic achievements. In practical teacher development and incentive mechanisms, the findings suggest that tailoring development plans to specific capabilities and potential can lead to more effective educational outcomes. It is recommended that universities allocate research resources based on evaluation results and establish plans for teachers’ career development and training.

### Discussion

In this study, the T-S fuzzy neural network model’s ability to handle both quantitative and qualitative data effectively addressed some of the critical gaps in traditional evaluation methods. This adaptability is particularly important in an era where educational standards and demands are constantly evolving. Moreover, the model’s reliance on a combination of expert input and robust data-driven analytics enhances its reliability and reduces the subjectivity often associated with educational evaluations.

Future research could refine the model by incorporating additional indicators, focusing on interdisciplinary contributions and international collaboration, which are increasingly important in global academia. Additionally, longitudinal studies could examine the sustainability of the model’s predictions over time, providing deeper insights into the effectiveness of development strategies based on this model.

By pushing the boundaries of traditional evaluation methods, this study contributes to a more flexible and comprehensive framework for assessing academic capabilities, potentially influencing policy decisions and resource allocation in higher education institutions.

### Application challenges

In the practical application of the T-S fuzzy neural network model to assess the sustainable research capabilities of university teachers, several multifaceted challenges are encountered.

Firstly, the real-time collection and processing of data present a significant issue. Information on university teachers’ research output, teaching activities, and interactions with practice needs to be tracked through continuously updated databases, demanding an efficient data processing system and assurance of data accuracy and completeness. How to swiftly and accurately collect these dynamically changing data and effectively integrate them into the model represents a key technical challenge.

Secondly, the adaptability and generalization ability of the model are crucial challenges in real-time applications. Given the substantial differences in research activities, teaching methods, and practical interactions among teachers from different universities and academic fields, the model must be capable of adapting to various backgrounds and conditions. Additionally, with changes in educational policies and research environments, the model needs constant adjustments and optimizations to maintain the accuracy and relevance of its evaluation results.

Furthermore, the interpretability and operability of the evaluation results in real-time applications must be considered. Providing valuable feedback and suggestions for university management and individual teachers, rather than just a set of abstract numbers, is crucial for enhancing the model’s practical application value.

Finally, privacy protection and data security are critical issues that cannot be overlooked in real-time applications. Ensuring the confidentiality of personal data and the security of data storage and transmission while collecting and processing information on university teachers’ research and teaching activities requires strict adherence to legal and regulatory requirements.

### Research limitations and prospects

This study has certain limitations that require improvement in the following areas:

Firstly, the issue of training sample selection. A primary condition for artificial neural networks to simulate biological neural networks for information processing is having a large amount of sample data. However, nearly all models and methods are implemented on small and limited datasets. Due to resource constraints, this study obtained only 126 valid data sets, using 90 of them as training samples. Quantitatively, the sample size is not large enough. To ensure representativeness, we selected university teachers from different levels as research samples, acknowledging that the sample data available for this study was indeed limited. In future research, it would be beneficial to increase the sample size and consider categorizing responses by academic discipline. This would allow for a more comprehensive verification of the representativeness of the disciplines and provide insights into specific characteristics of sustainable research capabilities across different academic fields.

Secondly, determining the relationship between the scores of various indicators and actual sustainable research capabilities is challenging. During network training, sensitivity analysis can be performed by adjusting the input values of each indicator by a small margin (e.g., ±0.1 points) to observe the impact of changes in indicator scores on the scores based on actual sustainable research capabilities. However, due to the complexity and non-linearity of the problem at hand, the results presented by artificial neural networks are generally non-parametric, meaning it is not possible to intuitively observe the quantitative relationship between independent and dependent variables as in econometric models. Furthermore, the study did not analyze the interplay between various indicators, which might be complemented by other research methods or tools in the future, providing more valuable references for evaluating university teachers’ sustainable research capabilities.

Lastly, the issue of network optimization. The fuzzy inference process of the T-S fuzzy neural network is essentially implemented through a BP neural network structure. Although the BP neural network has good fitting capabilities, it can produce significant errors in predicting some complex nonlinear problems. This could be addressed by optimizing the BP neural network using genetic algorithms, which optimize the initial weights and biases of the BP neural network to improve prediction accuracy. However, genetic algorithms have limitations; they cannot enhance the prediction accuracy of BP neural networks with large prediction errors. Especially for cases where the small number of samples leads to large prediction errors, using genetic algorithms to optimize the network’s prediction accuracy typically does not result in significant improvement. Other optimization techniques, such as particle swarm optimization or ant colony algorithms, could be considered for optimizing the initial weights of the fuzzy neural network to enhance its predictive capability. However, this step was not implemented in this study and is hoped to be explored in further in-depth research.
